# Analysis of BH3-only proteins upregulated in response to oxygen/glucose deprivation in cortical neurons identifies Bmf but not Noxa as potential mediator of neuronal injury

**DOI:** 10.1038/cddis.2014.426

**Published:** 2014-10-09

**Authors:** S Pfeiffer, U Anilkumar, G Chen, S Ramírez-Peinado, J Galindo-Moreno, C Muñoz-Pinedo, J H M Prehn

**Affiliations:** 1 Department of Physiology and Medical Physics, Royal College of Surgeons in Ireland, 123 St. Stephen's Green, Dublin 2, Ireland; 2 Cell Death Regulation Group, IDIBELL (Bellvitge Biomedical Research Institute), Gran Via de L'Hospitalet 199, Barcelona 08908, Spain

## Abstract

Stress signaling in response to oxygen/glucose deprivation (OGD) and ischemic injury activates a group of pro-apoptotic genes, the Bcl-2 homology domain 3 (BH3)-only proteins, which are capable of activating the mitochondrial apoptosis pathway. Targeted studies previously identified the BH3-only proteins Puma, Bim and Bid to have a role in ischemic/hypoxic neuronal injury. We here investigated the transcriptional activation of pro-apoptotic BH3-only proteins after OGD-induced injury in murine neocortical neurons. We observed a potent and early upregulation of *noxa* at mRNA and protein level, and a significant increase in Bmf protein levels during OGD in neocortical neurons and in the ipsilateral cortex of mice subjected to transient middle cerebral artery occlusion (tMCAO). Surprisingly, gene deficiency in *noxa* reduced neither OGD- nor glutamate-induced neuronal injury in cortical neurons and failed to influence infarct size or neurological deficits after tMCAO. In contrast, *bmf* deficiency induced significant protection against OGD- or glutamate-induced injury in cultured neurons, and *bmf*-deficient mice showed reduced neurological deficits after tMCAO *in vivo*. Collectively, our data not only point to a role of Bmf as a BH3-only protein contributing to excitotoxic and ischemic neuronal injury but also demonstrate that the early and potent induction of *noxa* does not influence ischemic neuronal injury.

Cerebral ischemia, resulting from occlusion or hemorrhaging of blood vessels supplying the brain, triggers a complex series of physiological, biochemical and gene expression changes ultimately mediating neuronal injury and activation of cell death mechanisms.^1^ Outside of the damaged necrotic infarct core following focal cerebral ischemia, the ischemic penumbra presents an area of less severe neuronal injury, functionally impaired but structurally intact, with active cell death pathways contributing to neuronal injury and loss of neurological function over time.^2^ Among pro-inflammatory and other processes, progressive neuronal injury of the ischemic penumbra is associated with glutamate-induced depolarization, energetic stress and activation of AMP-activated protein kinase (AMPK), with the later triggering both pro-survival and pro-apoptotic signaling in neurons.^3,4^

Mitochondrial-mediated apoptosis has been shown to be involved in neuronal cell death after cerebral ischemia in studies of both patient samples and animal models of acute stroke,^5^ with reduced expression of anti-apoptotic Bcl-2 and Bcl-w and induction of pro-apoptotic Bax observed within the ischemic core and surrounding penumbra.^6^ Translocation of cytosolic Bax to the mitochondrial outer membrane is key for the activation of mitochondrial apoptosis in neurons.^7, 8, 9, 10, 11^ This process is inhibited by anti-apoptotic Bcl-2 family proteins,^12, 13, 14, 15^ and overexpression of anti-apoptotic Bcl-2 family proteins have demonstrated *in vivo* neuroprotective roles against ischemic neuronal cell death.^15, 16, 17^ Bax translocation and membrane insertion eventually results in mitochondrial membrane permeabilization and the release of cytochrome *c* and other pro-apoptotic proteins that trigger caspase-dependent and -independent cell death processes.^18,19^ Bax activation is triggered by the transcriptional and posttranslational activation of Bcl-2 homology domain 3 (BH3)-only proteins that directly activate Bax and/or indirectly activate Bax by neutralizing the activity of anti-apoptotic Bcl-2 family proteins (‘de-repression').^19, 20, 21^

A role for several BH3-only proteins, in particular Bid .^22,23^ and Puma, .^24^ in ischemic neuronal injury has been previously suggested in studies using animals deficient in these genes. Among the pro-apoptotic BH3-only proteins implicated in ischemic neuronal cell death, the roles of Bcl-2-modifying factor (Bmf) and Noxa remain poorly investigated.^25^ Both are known to act as indirect activators of apoptosis with roles as ‘de-repressors', preventing sequestration of direct activators such as Puma, Bid and Bim by pro-survival Bcl-2 family with limited effect on cytochrome *c* release in cellular and isolated mitochondrial studies.^20,26,27^ Bmf has been reported to have roles in cell death in response to anoikis through the inhibition of Bcl-2 .^28,29^ and has been shown to be induced under conditions of hypoxia and through c-jun N-terminal kinase (JNK) and AMPK activation in response to bioenergetic stress,^30,31^ as well as having roles in autophagy and in cell death induced by inhibition of glucose metabolism.^32, 33, 34^ Noxa was originally described as a primary p53-response gene and mediator of p53-dependent apoptosis^27^ but can also be transcriptionally induced during ischemia through hypoxia-inducible factor (HIF)-1alpha,^35^ JNK and AMPK activation.^27,36^ This study investigated whether *noxa* and *bmf* are induced in response to oxygen/glucose deprivation (OGD) in cultured cortical neurons *in vitro* and in a mouse model of transient focal ischemic injury *in vivo* and investigates the role of these pro-apoptotic genes in mediating neuronal injury *in vitro* and *in vivo*.

## Results

### Levels of *noxa* mRNA are increased following OGD in primary cortical neurons

**Figure 1:**
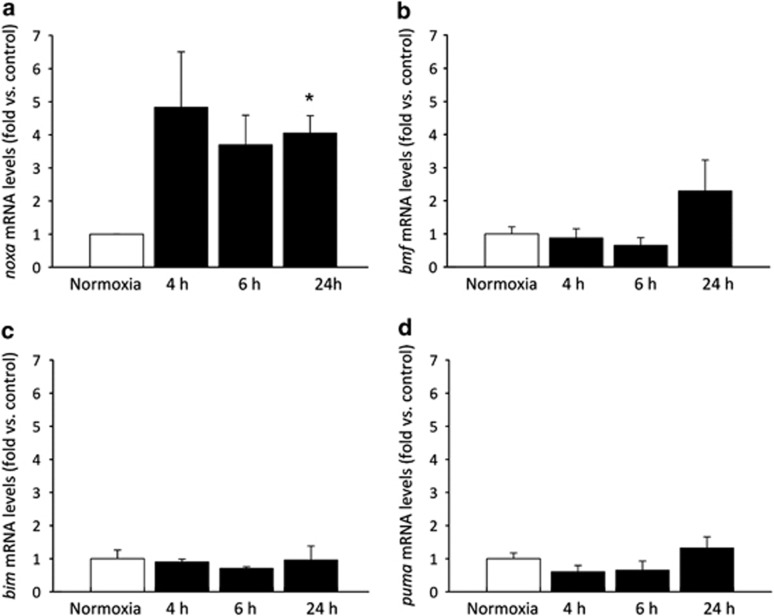
Induction of BH3-Only proteins in response to OGD in cortical neurons. Real-time qPCR analysis of mRNA expression of BH3-only proteins in cortical neurons subjected to 45 min OGD and allowed to recover under normoxic conditions for the times indicated. Controls were maintained under normoxic conditions. (**a**) mRNA levels for *noxa* were found to be upregulated from 4 h, and levels were maintained significantly up to 24 h. (**b**) *bmf* mRNA is not significantly induced by OGD. (**c** and **d**) Other markers investigated, *bim* and *puma*, respectively, showed no significant change when compared with control. Data were normalized to *β*-actin levels and expressed as relative to controls; presented as mean±S.E.M.; *n*=3 separate experiments carried out in triplicate. *P<0.05 compared with sham-treated control (ANOVA, *post hoc* Tukey's test)

In order to identify which pro-apoptotic BH3-only proteins may have a role in ischemic neuronal injury, cultures of neocortical neurons were subjected to OGD *in vitro.* Mature cultures of neocortical neurons were subjected to 45 min of OGD and allowed to recover under normoxic conditions for various timepoints (4, 6, 24 h) at which points mRNA levels of BH3-only proteins were assessed by real-time quantitative PCR (qPCR) analysis. mRNA levels for *noxa* were found to be upregulated from 4 h onward, and levels were maintained significantly up to 24 h at 3.9-fold (Figure 1a). Other markers investigated, including *bim* and *puma* (Figures 1b–d), showed no significant change when compared with controls. We also observed a delayed, 2.3-fold over-representation in *bmf* expression 24 h after OGD; however, this was not within the range of statistical significance.

### *noxa* and *bmf* mRNA changes following transient focal cerebral ischemia

**Figure 2:**
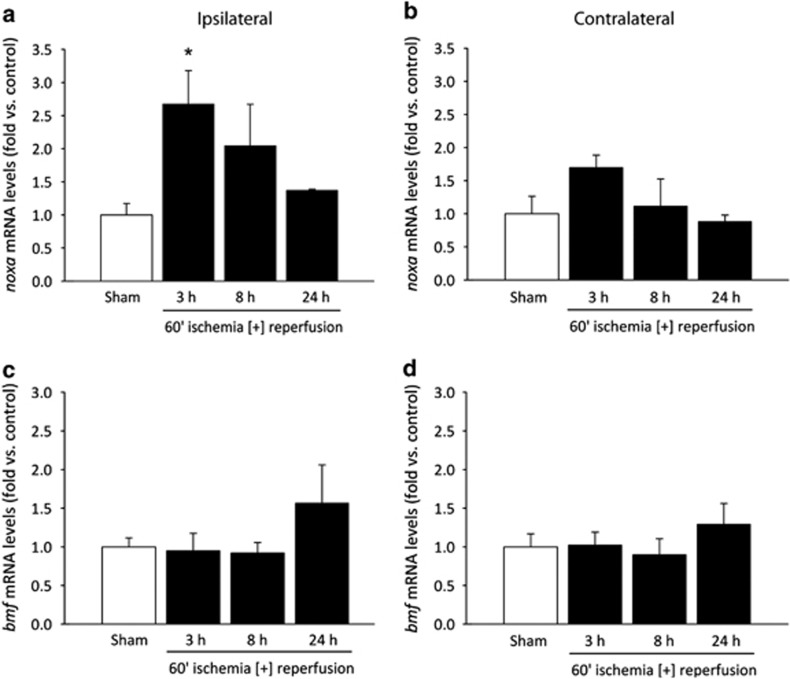
Induction of *bmf* and *noxa* following transient focal cerebral ischemia in wt mice. Real-time qPCR measurement of (**a** and **b**) *noxa* and (**c** and **d**) *bmf* mRNA expression in the cortex 3, 8 and 24 h following 60 min MCA occlusion with reperfusion. Data were normalized to *β*-actin levels and fold increases expressed relative to matched controls for *n*=3 per group. *P<0.05 compared with sham-treated control (ANOVA, *post hoc* Tukey's test)

Following the data obtained *in vitro* from neocortical neurons, we attempted to validate these results *in vivo* in a mouse model of transient focal ischemia. Wild-type (WT) mice were sham-treated or subjected to 60 min ischemia by occlusion of the middle cerebral artery (MCA) followed by reperfusion for 3, 8 and 24 h, at which time points mice were euthanized and brains were removed for gene expression analysis to determine the expression of *noxa* and *bmf* mRNA in the ipsilateral and contralateral cortex. Real-time qPCR analysis determined that *noxa* mRNA expression was significantly increased 2.7-fold in the ipsilateral cortex of animals 3 h following reperfusion and maintained a 2.1- and 1.4-fold increase at 8 and 24 h, respectively, returning towards baseline (Figure 2a), compared with ipsilateral cortices of sham-operated mice. Cortical expression of *bmf* mRNA was maintained at baseline, 3 and 8 h and increased 1.6-fold at 24 h following reperfusion after MCA occlusion in the ipsilateral cortex (Figure 2c) compared with sham-operated ipsilateral cortices, but again this did not reach the level of statistical significance. Contralateral cortical mRNA expression of mice subjected to transient middle cerebral artery occlusion (tMCAO) showed no significant difference to sham-operated contralateral cortices but tended to mirror the trends observed in the ipsilateral cortex (Figures 2b and d), consistent with a progressive increase of intracranial pressure due to cerebral edema. These data confirm *in vitro* observations of early *noxa* induction after ischemic conditions, maintained up to 24 h. In contrast, induction of *bmf* mRNA, similar to the OGD experiments, was not statistically significant following cerebral ischemia (Figure 2c).

### Deletion of *noxa* does not confer protection to excitotoxic and OGD-induced neuronal injury in cultured primary cortical neurons

**Figure 3:**
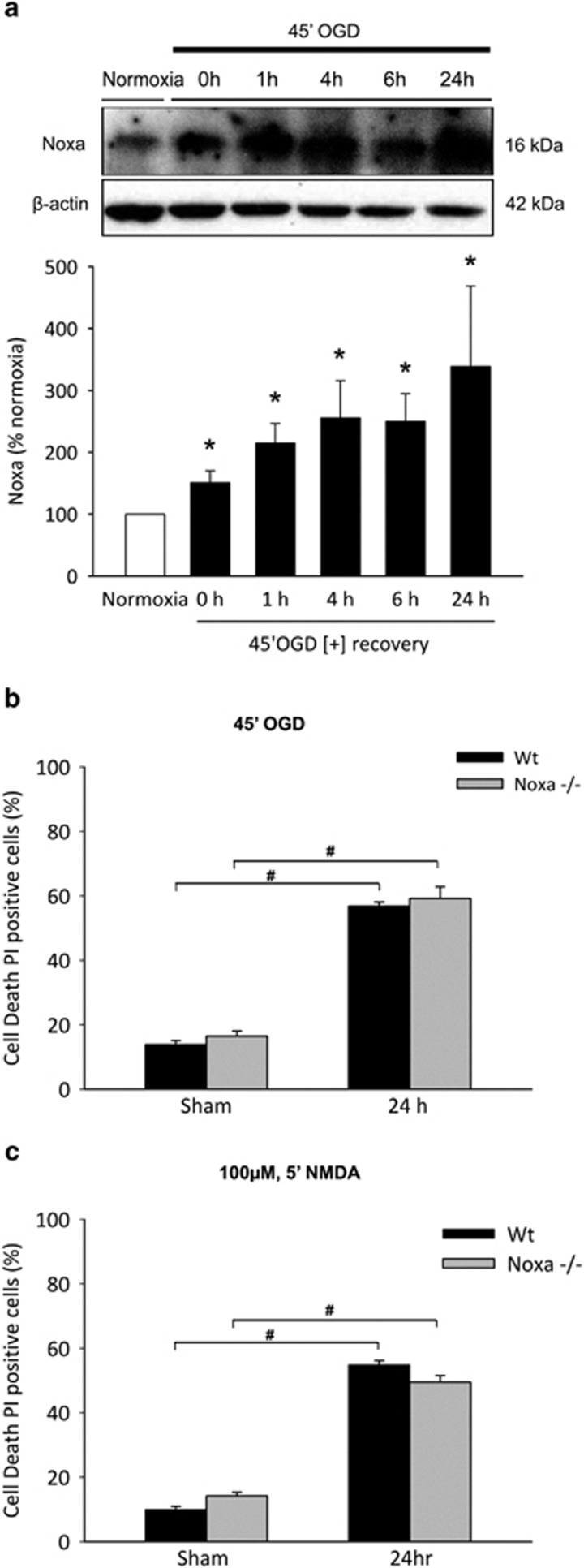
Induction of Noxa and effect of *noxa* deletion on neuronal injury primary cortical neurons. (**a**) Western blotting and densitometric analysis comparing the levels of Noxa induction in OGD-treated cortical neurons allowed to recover under normoxic conditions for the times indicated, confirming significant Noxa protein induction. Significant increases in Noxa induction were observed at 0 h and maintained up to 24 h compared with normoxic controls. Data presented as mean±S.E.M. from *n*=4 independent experiments from *n*=4 independent cultures. *P<0.05 compared with sham-treated controls. (**b** and **c**) Cortical neurons derived from WT and *noxa-*deficient mice were sham-treated or subjected to either 45 min OGD or a model of excitotoxic NMDA receptor overactivation by exposure to NMDA/glycine (100 *μ*M/10 *μ*M) for 5 min and allowed to recover under normoxic conditions for 24 h. Cell death was assessed after 24 h in each model by Hoechst and PI staining, and three subfields were captured per well, with a minimum of three wells analyzed per condition. Nuclei uniformly stained with Hoechst were counted as viable and condensed; PI-positive nuclei were scored as dead neurons and expressed as a percentage of the total population. Deficiency of *noxa* in cortical neurons neither reduced (**b**) OGD- or (**c**) glutamate-induced neuronal injury in cortical neurons. Data presented as mean±S.E.M., figures representative of *n*=3 independent experiments from *n*=3 independent cultures carried out in triplicate with similar results. *P<0.05 compared with sham-treated control; *^#^P*<0.05 between treatments (ANOVA, *post hoc* Tukey's test)

Early and significant induction of *noxa* mRNA observed both *in vitro* after OGD and *in vivo* in response to ischemia prompted investigation of Noxa protein induction and the effects of deletion of *noxa* in ischemic neuronal injury. Levels of Noxa were examined in WT primary cortical neurons after OGD treatment followed by recovery for 0, 1, 4, 6 and 24 h by western blotting. Controls were sham-exposed and maintained under normoxic conditions. Forty-five minutes of OGD in healthy primary cortical neurons was sufficient to induce a significant increased expression of Noxa protein from 0 h after treatment and maintained significantly up to 24 h (Figure 3a) compared with normoxic controls, consistent with the observed upregulation of *noxa* mRNA. As a positive control for OGD-induced neuronal injury, we observed a significant increase in the accumulation of caspase/calpain-mediated II-spectrin breakdown cleavage products^37, 38, 39^ in cortical neurons after OGD (see below; Figure 5b).

In order to examine the effects of deletion of *noxa* in ischemic neuronal injury, cortical neurons derived from WT and *noxa-*deficient mice were sham-treated or subjected to either 45 min OGD or exposure to *N*-methyl-D-aspartate (NMDA)/glycine (100 *μ*M/10 *μ*M) for 5 min and allowed to recover under normoxic conditions for 24 h. Controls were sham-exposed and maintained under normoxic conditions. Assessment of cell death 24 h after OGD treatment (Figure 3b) or NMDA treatment (Figure 3c) by Hoechst and PI staining indicated no difference in the cell death levels in WT cortical neurons subjected to OGD (56.82%±1.22) or NMDA (54.89%±1.31) insult compared with *noxa-*deficient cortical neurons subjected to OGD (59.17%±3.62) or NMDA (49.47%±2.03), demonstrating that no significant protection is conferred with deletion of *noxa in vitro*.

### *noxa*-deficient mice are not protected from ischemic injury *in vivo*

**Figure 4:**
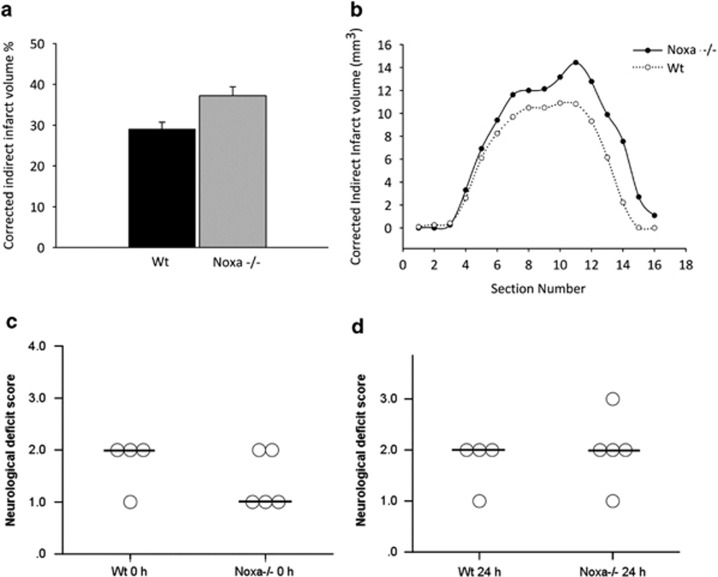
*noxa-*Deficiency confers no protection against ischemic injury. Infarct volume was assessed 24 h after focal cerebral ischemia by cresyl violet/Nissl staining. Infarct volume was calculated for direct infarct volume and corrected for edema of infarcted tissue for (**a**) total indirect infarct volume and (**b**) indirect infarct volume of each section. Mice deficient in *noxa* failed to influence infarct size compared with WT (Mann–Whitney *U* test). Results were quantified and presented as a percentage of infarct volume compared with WT treated. (**c** and **d**) Neurological deficit scores after induction of tMCAO in WT and *noxa-*deficient mice at 0 and 24 h following 60 min ischemia with reperfusion. No differences in neurological scoring outcome were found between *noxa-*deficient compared with WT matched controls after 24 h reperfusion. Circles and bar represent deficit score and median score, respectively. Data are representative of *n*≥4–5 for each group WT and *noxa-*deficient mice

Next we sought to determine a role for Noxa *in vivo* by ischemic infarct assessment in WT compared with *noxa-*deficient mice subjected to transient focal cerebral ischemia. We confirmed no significant difference in age and weight of the mice at the time of experiment and observed no significant differences between genotypes in the plasticity of the posterior communicating artery (PcomA; see Methods). Assessment of infarct volume was made 24 h following 60 min MCA occlusion with reperfusion by Cresyl violet/Nissl staining encompassing the cortex and striatum within the vascular territory of the MCA and expressed as a percentage of contralateral hemisphere to correct for differences in the brain size and brain edema. Mice deficient in *noxa* did not demonstrate any significant reduction in infarct volume (Figure 4a), with an infarct volume of 37.2±2.2% compared with 29.0±1.7% in WT animals (Figures 4a and b). Neurological deficit scores were also evaluated at 0 h (Figure 4c) and 24 h (Figure 4d) following ischemia with reperfusion. There was no significant improvement in neurological deficits observed in *noxa-*deficient mice at either time point examined. Taken together, the above data demonstrate that the early induction of *noxa* may contribute to non-excitotoxic components of neuronal injury but has no influence neuronal survival after OGD *in vitro* or ischemic injury *in vivo*.

### Bmf protein levels are increased following OGD in primary cortical neurons and focal cerebral ischemia in mice

**Figure 5:**
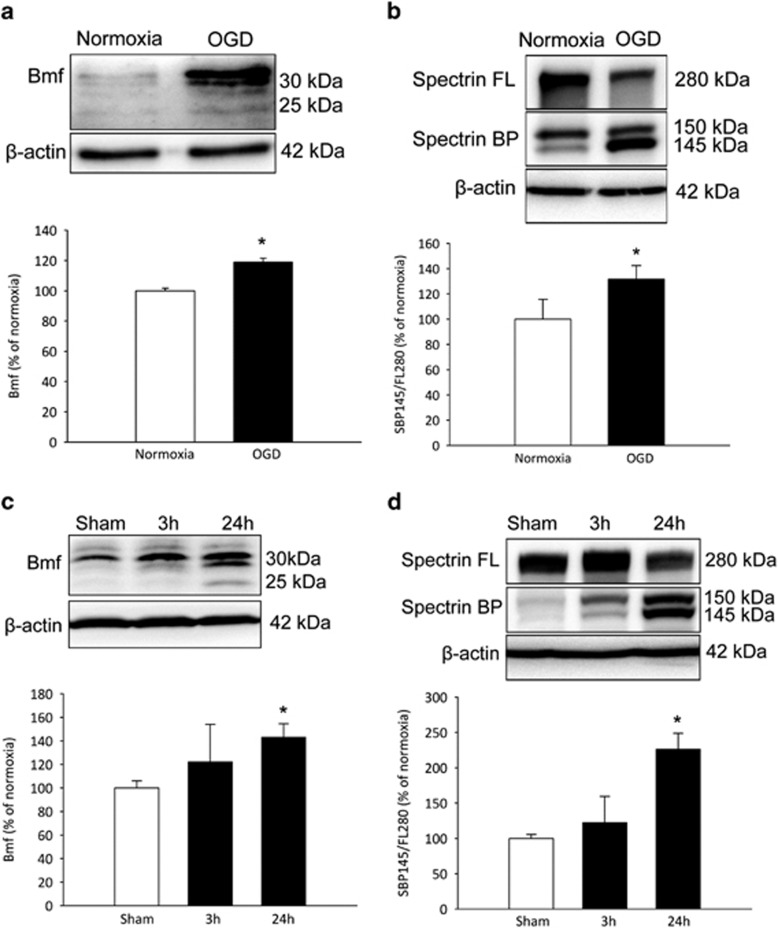
Increased Bmf protein expression following OGD in cortical neurons and transient focal cerebral ischemia in WT mice. (**a** and **b**) Western blotting and densitometric analysis comparing the levels of Bmf induction and Spectrin cleavage in cortical neurons, (**a**) confirming Bmf protein induction at 24 h and (**b**) demonstrating an increased accumulation of calpain/caspase-3-generated *α*II spectrin breakdown products (145/150 kDa) in OGD-treated cultures. Significant increases in Bmf protein induction and Spectrin cleavage were observed at 24 h compared with sham-treated cultures. Data presented as mean±S.E.M. from *n*=5 independent experiments from *n*=5 independent cultures carried out in triplicate. *P<0.05 compared with sham-treated controls. (**c** and **d**) Western blotting and densitometric analysis comparing levels of Bmf induction and Spectrin cleavage in the ipsilateral cortex 3 and 24 h following ischemia in WT mice, (**c**) confirming Bmf protein induction and (**d**) demonstrating an increased accumulation of *α*II spectrin breakdown products at 24 h reperfusion. Significant increases in Bmf protein induction and Spectrin cleavage were observed at 24 h compared with matched controls. Densitometry data are expressed as Bmf or as a ratio of the 145 kDa spectrin breakdown product (BP) and the 280 kDa full length (FL) protein normalized to *β*-actin. Data presented as mean±S.E.M. from *n*=4 per group. *P<0.05 compared with matched controls

To explore a possible increase in Bmf protein expression following ischemic neuronal injury, levels of Bmf were examined in primary cortical neurons after OGD treatment and in the ipsilateral cortex of WT mice subjected to 60 min ischemia followed by reperfusion for 3 and 24 h by western blotting. Forty-five minutes of OGD in healthy primary cortical neurons was sufficient to induce a significant increased expression of Bmf 24 h after treatment (Figure 5a). Expression of Bmf was also found to be significantly increased in the ipsilateral cortex of WT mice at 24 h following reperfusion after ischemia (Figure 5c).

To confirm initiation of apoptotic cell death resulting from OGD or ischemia, quantitative western blotting analysis was also carried out for *α*II-spectrin proteolysis. *α*II-Spectrin proteolysis during apoptosis triggered by cerebral ischemia has been implicated in hypoxic–ischemic neuronal injury.^37,40,41^ Considerable evidence documenting the accumulation of caspase-3- and calpain-mediated *α*II-spectrin breakdown products and corresponding decrease of full-length *α*II-spectrin protein after OGD in hippocampal slice cultures *in vitro* and in the ipsilateral cortex of rodents subjected to tMCAO injury *in vivo* provides substantial evidence supporting the use of *α*II-spectrin breakdown as a useful biomarker in models of focal ischemia.^37, 38, 39^ Western blotting analysis revealed a significantly increased accumulation of the 145 kDa *α*II-spectrin breakdown product both *in vitro* in cortical neurons (Figure 5b) and *in vivo* in the ischemic ipsilateral cortex (Figure 5d) at 24 h, indicating the initiation of calpain/caspase-3-dependent apoptosis as a result of OGD and focal cerebral ischemia.

### Cell death is attenuated in *bmf*-deficient primary cortical neuron cultures following OGD

**Figure 6:**
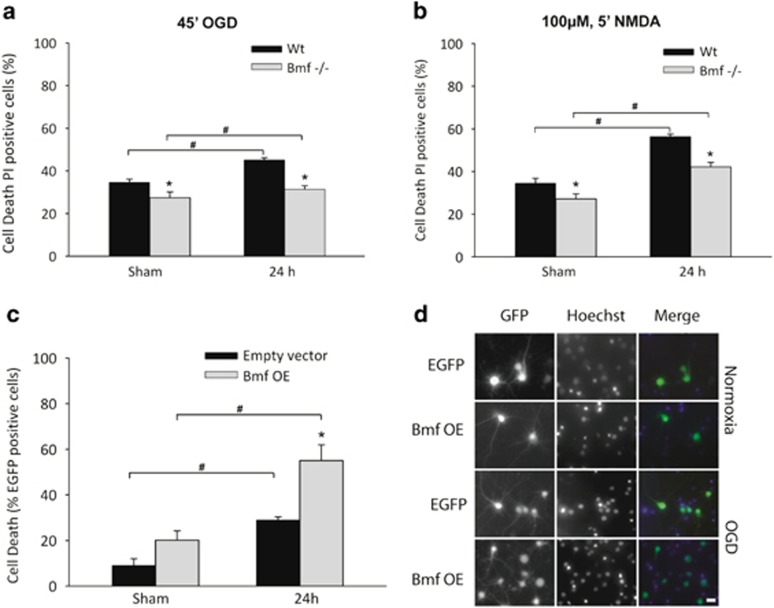
Deletion of *bmf* confers protection against neuronal injury induced by OGD in cultured primary cortical neurons. Cortical neurons derived from WT and *bmf-*deficient mice were sham-treated or subjected to either 45 min OGD or a model of excitotoxic NMDA receptor overactivation by exposure to NMDA/glycine (100 *μ*M/10 *μ*M) for 5 min and allowed to recover under normoxic conditions for 24 h. Cell death was assessed 24 h after OGD or NMDA treatment in each model by Hoechst and PI staining. (**a** and **b**) Cell death was significantly reduced in *bmf-*deficient neurons compared with WT, strongly implicating *bmf* in the apoptotic response to both (**a**) OGD and (**b**) excitotoxic NMDA receptor overactivation. Data presented as mean±S.E.M., figures representative of *n*=3 independent experiments from *n*=3 independent cultures carried out in triplicate with similar results. *P<0.05 compared with sham-treated control; *^#^P*<0.05 between treatments (ANOVA, *post hoc* Tukey's test). (**c**) Quantification of the effect of Bmf overexpression (OE) in cortical neurons on cell survival after OGD. Neurons were sham-treated or subjected to 45 min OGD and allowed to recover under normoxic conditions for 24 h. GFP and Hoechst 33358 (1 *μ*g/ml) fluorescence images were acquired to identify transfected cells and quantify nuclear apoptosis. The number of Hoechst-positive cells with condensed nucleus in eGFP-positive transfected neurons was quantified. Data presented as mean±S.E.M. from *n*=2 independent platings carried out in triplicate and pooled. *P<0.05 compared with sham-treated control; ^#^*P*<0.05 between treatments. (**d**) Refpresentative images of Bmf and eGFP co-transfected neurons and control eGFP transfected neurons sham-treated or subjected to 45 min OGD and allowed to recover under normoxic conditions for 24 h. Scale bar, 10 *μ*m

We also examined the effect of gene deficiency of *bmf* in *in vitro* models of neuronal injury, by OGD and excitotoxic NMDA receptor overactivation in cortical neurons derived from WT and *bmf-*deficient mice. Assessment of cell death 24 h after OGD treatment (Figure 6a) or NMDA treatment (Figure 6b) by Hoechst and PI staining indicated significantly reduced cell death in *bmf-*deficient neurons subjected to OGD (31.4±1.6%) or NMDA (42.2±2.0%) insult compared with WT cortical neurons subjected to OGD (45.2±1.0%) or NMDA (56.3±1.15%), implicating *bmf* in the apoptotic response to both OGD and NMDA receptor overactivation. In both experiments, there was no difference in cell death between WT and *bmf*-deficient cells for sham-treated controls.

In order to demonstrate that overexpression of *bmf* would reverse the neuroprotective effect observed in *bmf-*deficient neurons, cortical neurons derived from WT mice were co-transfected with a vector expressing *bmf* and an enhanced green fluorescent protein (eGFP)-expressing vector and subjected to OGD. Assessment of cell death of eGFP-positive cells 24 h after OGD treatment (Figure 6c) indicated that overexpression of *bmf* significantly exacerbated cell death when compared with control eGFP-transfected neurons.

### *bmf* may contribute to ischemic neuronal injury *in vivo*

**Figure 7:**
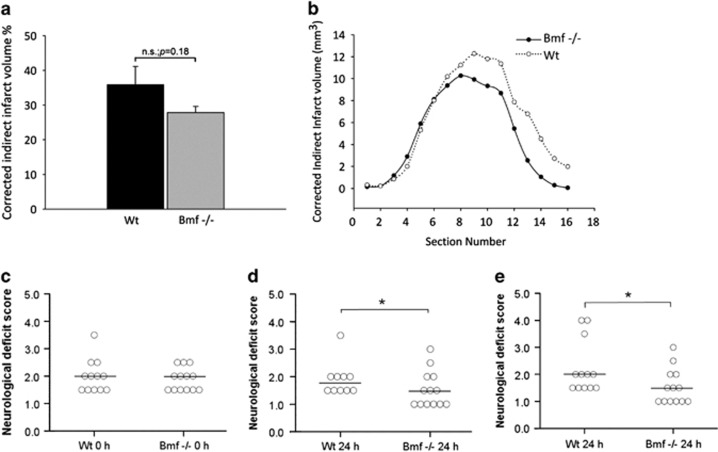
*bmf-*Deficiency confers protection against ischemic injury. Infarct volume was assessed 24 h after focal cerebral ischemia by cresyl violet/Nissl staining. Infarct volume was calculated for direct infarct volume and corrected for edema of infarcted tissue for (**a**) total indirect infarct volume and (**b**) indirect infarct volume of each section. Lower representation of infarct volume in mice deficient in *bmf* following tMCAO was not found to be significantly reduced when compared with WT mice (Mann–Whitney *U* test). Results were quantified and presented as a percentage of infarct volume compared with WT-treated mice. (**c** and **d**) Neurological deficit scores after induction of tMCAO in WT and *bmf-*deficient mice at 0 and 24 h following 60 min ischemia with reperfusion. Mice deficient in *bmf* score were significantly better than WT matched controls after 24 h reperfusion (Fisher's exact test). (**e**) Neurological deficit scores at 24 h following 60 min ischemia with reperfusion with inclusion of two WT mice that demonstrated stroke-related mortality before 24 h. The *bmf-* deficient mice maintained a significant reduced deficit compared with WT controls after 24 h reperfusion. Circles and bar represent deficit score and median score, respectively; *P<0.05 compared with WT control. Data are representative of *n*≥12–13 for each group of WT and *bmf-*deficient mice

We then sought to examine the effect of *bmf* deletion *in vivo*, comparing infarct volume in WT mice to *bmf-*deficient mice after tMCAO. Again, no significant difference in age and weight of the mice at the time of experiment was confirmed, and plasticity of the PcomA was observed (see Methods). Assessment of infarct volume revealed mice deficient in *bmf* demonstrated no significant difference in infarct volume following tMCAO (Figures 7a and b) when compared with WT. The infarct volume in *bmf*-deficient mice was 27.9±1.8% compared with 35.9±5.2% in WT mice; however, this did not reach the level of statistical significance.

Decreased neurological deficit scoring was also observed at 0 h (Figure 7c) and 24 h (Figure 7d) following ischemia with reperfusion, with *bmf-*deficient mice (Median (interquartile range), 1.5 (1.0)) showing improved deficits compared with WT mice (Median (interquartile range), 1.75 (0.5)) at 24 h (Figure 7d; *P*<0.05). Two WT mice showed stroke-related mortality before 24 h. Inclusion of these mice resulted in a neurological deficit score of 1.5 (1.0) (Median (interquartile range)) in *bmf*-deficient mice compared with 2.0 (1.63) (Median (interquartile range)) in WT mice, which again was statistically significant (Figure 7e). Collectively, this data indicate that deletion of *bmf* confers protection against OGD in primary cultured cortical neurons and suggests that *bmf* may contribute to ischemic neuronal injury and neurological deficit outcome *in vivo*.

## Discussion

Hypoxic–ischemic brain injury and associated cellular events are worthy of particular investigation as a well-known cause of neuronal cell death and damage. Pathophysiological analysis after focal brain ischemia reveals a necrotic core area of irreversible cell death resulting from total bioenergetic failure due to dramatically reduced metabolic rates of oxygen and glucose. Surrounding this necrotic core lies the ischemic penumbra, an area of less severe damage, functionally impaired but metabolically active tissue, balanced precariously between a complex schism of endogenous neuroprotective and neurotoxic events.^1^ Elucidation of the triggers and mediators of ischemic cell death mechanisms may provide gene-targeted intervention for neuroprotection in stroke, preventing ongoing injury and infarct progression.

To this end, our data from the present study indicate that the pro-apoptotic gene, *noxa*, although significantly induced, may possibly not become a relevant therapeutic target for the treatment of ischemic stroke. Using gene expression analysis, we observed a robust early induction of *noxa* mRNA and a corresponding significant induction of Noxa protein levels during OGD in cortical neurons. The transcriptional upregulation of *noxa* observed by conditions of OGD *in vitro* and focal ischemia *in vivo* is consistent with results reporting induction of *noxa* during hypoxia and after transient ischemia *in vivo* by Hif1*α* as a mediator of hypoxic cell.^25,35^ Hence the dependence of Noxa induction is not restricted to p53 and associated regulators;^27,36^ *noxa* transcription and expression can be activated by diverse apoptotic signals in both a p53-dependent and independent manner, with numerous regulators and control mechanisms that may be activated to induce *noxa* at the transcript level,^36^ leading to strong upregulation of *noxa* mRNA via multiple pathways as observed in our study. Surprisingly, however, our experiments revealed that gene deficiency in *noxa* did not protect against OGD-induced injury *in vitro* and that *noxa-*deficient mice were as susceptible to ischemic damage as WT littermates. Interestingly, Kim *et al.* .^35^ demonstrated a reduction of infarct volume in a rat model of focal ischemia by suppression of *noxa* expression by antisense oligonucleotides. Disparity in infarct volumes across models may be a result of experimental design and variation^42^ or could be due to species differences. It should also be noted that, despite successful applications of antisense strategies in gene silencing, the use of antisense oligonucleotides as an alternative to gene-knockout models *in vivo* in practice should be performed with adequate controls to ascertain whether observed physiological effects are the result of an antisense mechanism and not from non-specific effects, such as an intrinsic activity of the phosphorothioate backbone.^43^

Noxa is known to be poorly apoptotic in some cell types,^21,36^ and compared with other BH3-only proteins, Noxa demonstrates tethered potential for neutralization of pro-survival Bcl-2 proteins, with a more restricted role binding only Mcl-1 and A1.^20,21^ In this context, it is interesting to note that targeting of Mcl-1 by transgenic overexpression of Noxa failed to induce significant cell death in adenovirus oncoprotein E1A immortalized MEFs or NIH3T3 fibroblasts.^26^ Indeed, in a comparison of DNA-damage-induced apoptosis in mice lacking *puma* and/or *noxa*, damage was observed to be cell-type dependent; in mature CD4^+^ and CD8^+^ T cells and macrophages, Puma alone was sufficient to induce apoptosis, while loss of Noxa alone did not notably protect B or T cells.^44^ Puma was demonstrated to have a major role in most cell types studied, whereas Noxa was only seen to contribute in certain cell types. Contribution of Noxa to cell death may require cell-type dependent engagement of other additional pro-apoptotic factors. Although our analysis has attempted to exclude potentially mitigating factors, cerebrovascular development, subtle anatomical or physiological differences could contribute to our results. However, our data imply that the net effect of *noxa* is unlikely to contribute significantly to neuronal death signaling after cerebral ischemia.

In contrast to *noxa*, we did not observe a significant induction of *bmf* mRNA after OGD or cerebral ischemia. Subsequent experiments revealed a significant induction of Bmf protein levels following OGD in cortical neurons and after ischemic injury *in vivo*. Of note, experiments performed in primary cortical neurons indicated a significant protection in *bmf*-deficient neurons both *in vitro* after OGD- and NMDA-induced excitotoxic injury. In contrast, overexpression of Bmf resulted in significantly exacerbated cell death. *bmf* deficiency also demonstrated significantly reduced neurological scores after ischemic stroke in an *in vivo* model of tMCAO in mice. Clearly, it is possible that other BH3-only proteins such as Bid, Bim and Puma may compensate for a *bmf*-deficiency *in vivo*.^22,23,44,45^ Of note, *bmf* mRNA did not appear to be transcriptionally induced early in response to injury. This suggested that *bmf* contributes to neuronal injury via posttranscriptional activation mechanisms. Bmf is sequestered to the actin cytoskeleton-based myosin V motor complex through its interaction with dynein light chain (DLC)-2 and is released in response to stress stimuli to stimulate apoptosis.^28^ The DLC-binding motif in Bmf closely resembles the region in Bim that mediates its binding to DLC-1,^46,47^ and this interaction with DLC-2 negatively regulates the pro-apoptotic activity of Bmf;^28^ the localization of Bim and Bmf to the microtubules and actin cytoskeleton, respectively, may be determined fundamentally by their respective DLC partners. Additionally, Bmf has been shown to be induced posttranscriptionally through enhanced translation under conditions that cause repression of the CAP-dependent translation machinery, including hypoxia.^30^

Several BH3-only proteins, such as Bid, Bim and Bad,^22,48,49^ are also constitutively expressed in the brain and rapidly activated in response to focal cerebral ischemia. Collectively, these results suggest that *bmf* activation may contribute to the development of ischemic neuronal injury; however, there is likely a functional redundancy observable in BH3-only proteins contributing to ischemic neuronal injury, with similar lack of protection/tendency towards protection observed with deletion of *puma* in focal cerebral ischemia.^24^ Investigation of ischemic damage in BH3-only protein double-knockout models may be valuable to determine whether conductive actions of Noxa or Bmf with other BH3-only proteins occur, as reported in other cell death models.^50^

Collectively, the present study not only provides evidence for a role of Bmf as a BH3-only protein contributing to hypoxic/ischemic neuronal injury but also demonstrated that the early induction of *noxa* did not influence neuronal survival or ischemic injury, suggesting functional redundancy among BH3-only proteins for ischemia-induced neuronal death or functions of *noxa* independent of cell death signaling. These findings are important for future target selection strategies such as neuroprotective interventions to combat ischemic brain injury.

## Materials and Methods

### Gene-targeted mice

Animal experiments were carried out under license from the Department of Health and Children (Ireland) and in accordance with European Communities Council Directive (86/609/EEC). All procedures were reviewed and approved by local Research Ethics Committee of the Royal College of Surgeons in Ireland. For the analysis of *bmf* and *noxa* expression, targeted *bmf* and *noxa* mutant mice originally generated from C57BL/6-derived Bruce4 ES cells backcrossed onto a C57Bl/6J background were provided by Professor Andreas Strasser (WEHI, Melbourne, Australia) and bred as homozygous knockout colonies.^32,51^ WT C5Bl/6J mice were obtained from the Jackson Laboratories (Bar Harbor, ME, USA) and were backcrossed with *bmf* and *noxa* WT littermates for >10 generations for comparison to *bmf* and *noxa*-deficient mice

### Genotype analysis

WT and knockout alleles for *bmf* and *noxa* were confirmed by PCR analysis of genomic DNA extracted from tail snips using High Pure PCR Template Preparation Kit (Roche, Basel, Switzerland). Genotyping was performed using the specific primers: 5′-GGAGTTCAGACTTCGCCGAGAG-3′, 5′-GGCTGGTCACAAAGTTTGACACTG-3′ (WT allele-specific); 5′-GGAGTTCAGACTTCGCCGAGAG-3′, and 5′-GCAAGAGGCAAGCCCTTCACTTGG-3′ (mutant allele-specific) for *bmf*; and 5′-GGAGGGCATAAATGGGCAATGACAC-3′ (common), 5′-GATGCTTCTTGGGTGCACCCACAC-3′ (WT allele-specific reverse), and 5′-AAAGCAATCCCAAACGAC-3′ (mutant allele-specific reverse) for *noxa*.

### Preparation of primary mouse neocortical neurons and cell culture

Primary cultures of murine neocortical neurons were prepared and cultured as described previously^52^ with modifications. Briefly, hysterectomies of embryonic day 16–18 pregnant female WT, *bmf-* and *noxa-*deficient mice were carried out following cervical dislocation and embryonic cerebral cortices transferred to dissection medium on ice (PBS with 0.25% glucose and 0.3% BSA). The tissue was incubated in 0.25% trypsin-EDTA for 15 min at 37 °C, and trypsinization was stopped using media containing sera. Neurons were dissociated by gently pipetting and centifugated at 300 × *g* for 3 min, and the media containing trypsin was aspirated. Neocortical neurons were triturated in fresh plating medium (MEM containing 5% fetal bovine serum, 5% horse serum, 100 U/ml penicillin/streptomycin, 0.5 mM L-glutamine and 0.6% D-glucose). Cells were plated on poly-D-lysine-coated plates at 2 × 10^5^ cells/cm^2^ and maintained at 37 °C and 5% CO_2_ humidified atmosphere. After 24 h, medium was exchanged for 1 : 1 plating medium and feeding medium (Neurobasal media containing 100 U/ml penicillin/streptomycin, 2% B27 and 0.5 mM L-glutamine) containing 600 nM cytosine arabinofuranoside. At DIV (days *in vitro*) 3, medium containing cytosine arabinofuranoside was exchanged for fresh feeding media. All *in vitro* experiments were performed on mature cultures at DIV 8–12.

### Plasmids and transfection

Primary cortical neurons (DIV 10) were transfected using calcium phosphate.^53^ For overexpression of *bmf*, cells were transfected with a plasmid encoding murine *bmf* (generously provided by Professor A Villunger, Innsbruck Medical University, Innsbruck, Austria).^30^ A plasmid with enhanced GFP (eGFP-N1; Clontech, Saint-Germain-en-Laye, France) was used to allow the identification of transfected neurons for cell death assays. Cells were used for experiments 48 h after transfection.

### Oxygen–glucose deprivation

Healthy primary cortical neuron cultures at DIV 8–9 transferred to a hypoxic chamber (COY Lab Products, Grass Lake, MI, USA) with an atmosphere comprising 1.5% O_2_, 5% CO_2_ and 85% N_2_, with temperature maintained at 35 °C. Feeding medium was removed from cultures and replaced with preequilibrated, deoxygenated OGD medium bubbled with N_2_ for 1 h, consisting of (in mM): 0.3 CaCl_2_, 70 NaCl, 5.25 NaHCO_3_, 70 KCl, 1.25 NaH_2_PO_4_, 2 MgSO_4_, 10 sucrose, pH 6.8. After 45 min of OGD, cultures were returned to oxygenated feeding medium and allowed to recover for 24 h under normoxic conditions (21% O_2_ and 5% CO_2_). Sham-treated cultures were transferred to oxygenated glucose-free medium consisting of (in mM): 2 CaCl_2_, 125 NaCl, 25 NaHCO_3_, 2.5 KCl, 1.25 NaH_2_PO_4_, 2 MgSO_4_, and 10 sucrose, pH 7.5 and maintained under normoxic conditions (21% O_2_ and 5% CO_2_).^54^ Control cultures for protein and gene expression analysis were maintained under normoxic conditions.

### NMDA toxicity and determination of neuronal injury

Cortical neurons cultured on 24-well plate for DIV 8–9 were sham-treated or subjected to a model of excitotoxic injury induced by NMDA receptor overactivation^37^ by exposure to NMDA/glycine (100 *μ*M/10 *μ*M) for 5 min and washed twice in experimental buffer containing (in mM): 120 NaCl, 3.5 KCl, 0.4 KH_2_PO_4_, 20 HEPES, 5 NaHCO_3_, 1.2 Na_2_SO_4_, 1.2 CaCl_2_, and 15 glucose, pH 7.4, supplemented with high Mg^2+^ (1.2 mM).

Neuronal injury resulting from OGD, NMDA excitation or Bmf overexpression was assessed after 24 h in each model by staining neocortical neurons live with Hoechst 33258 at a final concentration of 1 *μ*g/ml and propidium iodide (PI) (Sigma, St. Louis, MO, USA) dissolved in culture medium after 24 h for 10 min at 37 °C. After incubation, nuclear morphology was assessed using an Eclipse TE 300 inverted microscope (Nikon Instruments, Amsterdam, Netherlands) and × 20 NA 0.45 dry objective. Images were taken using a SPOT RT SE CCD camera (SPOT Imaging Solutions, Sterling Heights, MI, USA) and the appropriate filter sets. For each time point and treatment, nuclei uniformly stained with Hoechst were counted as viable and condensed, and PI-positive nuclei were scored as dead neurons and expressed as a percentage of the total population, three subfields captured per well, with a minimum of three wells analyzed per condition. Images were processed using NIH Image J (Wayne Rasband, National Institute of Health, Bethesda, MD, USA). All scoring was carried out in a blinded manner.

### Gene expression analysis using real-time RT-PCR analysis

Total RNA was extracted from primary cortical neurons after OGD treatment *in vitro* or from murine cerebral cortex 3, 8 and 24 h after 60 min transient MCA occlusion using the RNeasy Mini Kit (Qiagen, Hilden, Germany). First-strand cDNA synthesis was performed using 1 *μ*g of total RNA as template and reverse transcribed using Superscript II (Invitrogen, Waltham, MA, USA) primed with 50 pmol random hexamers. Quantitative real-time PCR was performed using the Applied Biosystems 7500 Real-Time PCR System in a MicroAmp optical 96-well reaction plate with optical covers (Applied Biosystems, Waltham, MA, USA) and the QuantiTech SYBR green PCR kit (Qiagen) according to the manufacturer's instruction. Sense and antisense primers used were as follows: 5′-CAACACAAACCCAAGTCCT-3′ and 5′-CATTTGCAAACACCCTCCTT-3′ for *bim*; 5′-CCCATAAGCCAGGAAGACAA-3′ and 5′-CTGAAGCTTTCTGGCGATCT-3′ for *bmf*; 5′-TCAGGAAGATCGGAGACAAA-3′ and 5′-TGAGCACACTCGTCCTTCAA-3′ for *noxa*; 5′-ATGGACTCAGCATCGGAAGG-3′ and 5′-TGGCTCATTTGCTCTTCACG-3′ for *puma*; and 5′-GGGTGTGATGGTGGGAATGG-3′ and 5′-GGTTGGCCTTAGGGTTCAGG-3′ for *β-*actin. The data were analyzed using the Analysis AB 7500 SDS Software (Applied Biosystems), and the generation of specific PCR product was confirmed by melting curve analysis. Data were presented as mean±S.E.M.; all samples were normalized to *β-*actin levels and expressed as *n*-fold expression relative to matched controls for *n*=3 separate experiments carried out in triplicate.

### Protein extraction and western blotting

Tissue and cell pellets were lysed in ice-cold radio immunoprecipitation assay buffer (25 mM Tris HCl, 150 mM NaCl, 1% NP40, 1% sodium deoxycholate and 0.1% sodium dodecyl sulfate) followed by sonication of tissue on ice. Lysates were centrifuged at 10 000 × *g* for 10 min at 4 °C, and supernatants were used for western blotting. Resulting membranes were probed with a mouse monoclonal *α*-fodrin (*α*II-Spectrin) antibody (clone AA6; Millipore, Billerica, MA, USA, 1 : 1000), rat anti-Bmf monoclonal antibody (a gift from A Villunger, Innsbruck Medical University, Innsbruck, Austria; 1 : 500), rabbit anti-Noxa polyclonal antibody (ab23563; Abcam, Cambridge, UK, 1 : 2000) or a mouse monoclonal anti-*β*-actin (Sigma, 1 : 5000). Species-specific horseradish peroxidase-conjugated secondary antibodies (Pierce, Rockford, IL, USA, 1 : 5000) were detected using the Super-Signal West Pico Chemiluminescent Substrate (Pierce) and imaged using a FujiFilm LAS-3000 imaging system (FujiFilm, Sheffield, UK).

### Evaluation of PcomA plasticity

Evaluation of the PcomA plasticity was carried out in male WT, *bmf-* and *noxa-*deficient mice aged 8–10 weeks (20–24 g) as described^55^ without ischemia. Briefly, animals were anesthetized with Pentobarbital Sodium (Dolethal; 200 mg/kg), and transcardial perfusion fixation was performed with 20 ml 10 U/ml heparin in Ringer's solution and 20 ml 4% PFA followed by Cresyl Violet (5%, 1 ml). The plasticity of the bilateral PcomA was evaluated independently by a blinded investigator (GC) using a dissecting microscope and graded on a qualitative scale of 0–3 as follows: Score 0, no anastomosis between posterior cerebral artery (PCA) and superior cerebellar artery (SCA); Score 1, anastomosis between PCA and SCA in capillary phase; Score 2, small truncal PcomA; and Score 3, truncal PcomA. Statistical significant differences were analyzed using non-parametric statistics; no significant difference in PcomA plasticity was observed in *bmf-* or *noxa-*deficient *versus* WT control mice, *n*>6 per genotype (Fisher's exact test). The median score of PcomAs were (Median (interquartile range)) 1.0 (0.75) for *noxa-*deficient mice, 2.0 (0)) for *bmf-*deficient mice and 1.0 (1.75) for WT mice.

### Focal cerebral ischemia model

Induction of transient focal cerebral ischemia was performed in male WT, *bmf-* and *noxa-*deficient mice aged 8–10 weeks (20–24 g) using the monofilament suture method as described^56,57^ with modifications. Briefly, mice were anesthetized with 5% isoflurane 30% O_2_ and 65% N_2_O and maintained with 2% isoflurane, 33% O_2_ and 65% N_2_O for the duration of surgery (<20 min). Body temperature was maintained normothermic at 36.8–37.4 °C via a feedback-controlled heat blanket. A silicon-coated 8-0 nylon monofilament with rounded tip was introduced into the left internal carotid artery and advanced past the carotid artery bifurcation to occlude the MCA. After 60 min, the suture was removed to allow reperfusion. To control for effects of occlusion, sham-treated mice underwent the same surgical procedure, but the filament was not advanced to occlude the vessel. Effective occlusion and assessment of microcirculatory function was monitored by laser-Doppler flowmetry with a probe fixed to the exposed left parietal skull for continuous monitoring of regional cerebral blood flow (Perimed 5001 Master, Perimed, Järfälla, Sweden). Mice were euthanized 3, 8 and 24 h after reperfusion, and the brains were processed either for *bmf* and *noxa* expression analysis or for calculation of infarct volume. All surgical procedures were carried out in a blinded manner.

### Determination of ischemic infarct volume

Infarct volume was assessed 24 h after ischemia by a blinded investigator; 10 *μ*m coronal sections (*n*=16) from each brain were cut by Leica CM1950 Cryostat (Leica Biosystems Nussloch GmbH, Heidelberger Str, Germany) and taken at 500*μ*m intervals. Sections were stained with Cresyl violet/Nissl, and infarct area was evaluated quantitatively using an image analysis system by a blinded investigator (Leica Application Suite V3, Germany). Lesion volume was calculated as previously described^57^ by the infarct area multiplied by the section thickness and summed over the entire brain for direct infarct volume and corrected for edema of infarcted tissue for indirect infarct volume. Data are expressed as the percentage of contralateral hemisphere to correct for differences in the brain size and brain edema.

### Neurological deficit

Neurological deficit scores were assessed as described previously.^22^ Briefly, the neurological function of mice was evaluated for severity by a blinded investigator (GC) at 0 or 24 h after ischemia using a five-point scale, as follows: Score 0, no deficit; Score 1, weakness of the contralateral forepaw; Score 2, circling; Score 3, loss of righting reflex; and Score 4, no motor activity. In a separate analysis, stroke-induced death of the animal was scored as 5.

### Statistical analysis

Statistics were carried out on an SPSS-IBM software (IBM, Armonk, NY, USA). mRNA and densitometry data are presented as mean±S.E.M. and were analyzed by using one-way ANOVA followed by Tukey's *post hoc* test to determine statistical significance. *P*-values <0.05 were considered to be statistically significant. Infarct volumes were analyzed using non-parametric analyses (Kruskal–Wallis non-parametric analysis, Mann–Whitney *U*-test); Fisher's exact test was used to compare significant differences in PcomA plasticity and neurological deficit scores. Significance was accepted at *P*<0.05.

## Abbreviations

AMPK: AMP-activated protein kinase
BH3: Bcl-2 homology domain 3
Bmf: Bcl-2-modifying factor
DIV: days *in vitro*
eGFP: enhanced green fluorescent protein
NMDA: *N*-methyl-𝒟-aspartate
OGD: oxygen/glucose deprivation
PCA: posterior cerebral artery
PcomA: posterior communicating artery
qPCR: quantitative PCR
SCA: superior cerebellar artery
tMCAO: transient middle cerebral artery occlusion
WT: wild type
